# A Bidirectional Relationship Between Spontaneous Coronary Artery Dissection and Stress-Induced Cardiomyopathy in a Perimenopausal Woman: A Case Report

**DOI:** 10.7759/cureus.116673

**Published:** 2026-09-21

**Authors:** Nitya Panyala, Mohammad Freihat, Amanjit Singh, Manuel Rivera

**Affiliations:** 1 Internal Medicine, Ball Memorial Hospital, Indiana University School of Medicine, Muncie, USA; 2 Internal Medicine, Ball Memorial Hospital, Muncie, USA; 3 Cardiology, Ball Memorial Hospital, Muncie, USA

**Keywords:** acute st-elevation myocardial infarction, peri-menopause, spontaneous coronary artery dissection, stress induced cardiomyopathy, takotsubo cardiomyopathy

## Abstract

Spontaneous coronary artery dissection (SCAD) and stress-induced cardiomyopathy (SICM) are non-atherosclerotic causes of acute coronary syndrome that predominantly affect women. Their overlapping clinical, electrocardiographic, biomarker, and imaging features can make diagnosis challenging, and although their coexistence is increasingly recognized, the relationship between these entities remains poorly understood.

A 48-year-old female with cardiovascular risk factors presented with back and scapular pain and ST-segment elevations. Coronary angiography revealed Yip-Saw Class 2 SCAD in the mid-distal LAD with preserved flow. Echocardiography demonstrated wall motion abnormalities extending beyond the LAD territory with a reduced ejection fraction and apical ballooning suggesting concurrent SICM.

This case illustrates the concept that SCAD and SICM are important non-atherosclerotic causes of acute coronary syndrome in women and may present with overlapping features that complicate diagnosis. Their coexistence is biologically plausible given their marked female predominance and shared susceptibility related to estrogen-mediated vascular protection, which may be diminished during the perimenopausal transition. Clinicians should consider concurrent SICM when evaluating patients with SCAD, particularly when wall motion abnormalities extend beyond the affected coronary distribution.

## Introduction

Spontaneous coronary artery dissection (SCAD) and stress-induced cardiomyopathy (SICM) are important causes of acute coronary syndrome in women and may present with similar clinical, electrocardiographic, and imaging features, leading to diagnostic uncertainty. SCAD is an important non-atherosclerotic cause of myocardial infarction in younger and middle-aged women [1], while SICM is characterized by transient ventricular dysfunction frequently associated with emotional or physical stress [2]. The coexistence of these conditions can further complicate differentiation between ischemic myocardial injury and stress-mediated ventricular dysfunction.

However, recent literature suggests a potential bidirectional relationship between SCAD and SICM, in which either condition may precede or precipitate the other [3].

In this context, stress-induced myocardial dysfunction may contribute to increased coronary wall stress and subsequent dissection, whereas SCAD-related ischemia may trigger catecholamine-mediated myocardial stunning, resulting in SICM [4].

We present a case demonstrating the simultaneous presence of angiographic findings consistent with SCAD and echocardiographic features in keeping with SICM, highlighting the diagnostic challenge in determining the primary event. This overlap underscores the limitations of current diagnostic approaches and emphasizes the importance of integrating multimodal data when evaluating non-atherosclerotic acute coronary syndromes in women.

## Case presentation

A 48-year-old woman presented to an outside hospital with acute onset of left shoulder pain. Her medical history was notable for active tobacco use, hypertension, dyslipidemia, and pre-diabetes. She had no known history of coronary artery disease or prior cardiac events.

Her presentation was preceded by several days of anxiety and palpitations, which she attributed to work- and home-related stress. On initial evaluation, vital signs were stable, and physical examination was unremarkable. A 12-lead electrocardiogram demonstrated anterolateral ST-segment elevations, prompting emergent transfer to our facility for cardiac catheterization. Serial high-sensitivity troponin levels were 33,192 ng/L, 109,820 ng/L, and 20,270 ng/L (reference range ≤ 20 ng/L). B-type natriuretic peptide was 652 pg/mL (reference range ≤100 pg/mL).

The differential diagnosis included acute coronary artery occlusion, coronary artery dissection, SICM, and aortic dissection. Upon arrival, she underwent emergent coronary angiography, which demonstrated a long, smooth, and diffuse narrowing of the mid-to-distal left anterior descending (LAD) artery that persisted after administration of intracoronary vasodilators. Distal coronary flow was preserved. Angiographic findings revealed a long segment of diffuse luminal narrowing without focal stenosis (Figure 1, left panel). The angiographic appearance was consistent with Yip-Saw Type 2 SCAD, characterized by diffuse tubular narrowing without a discrete intimal flap.

**Figure 1:**
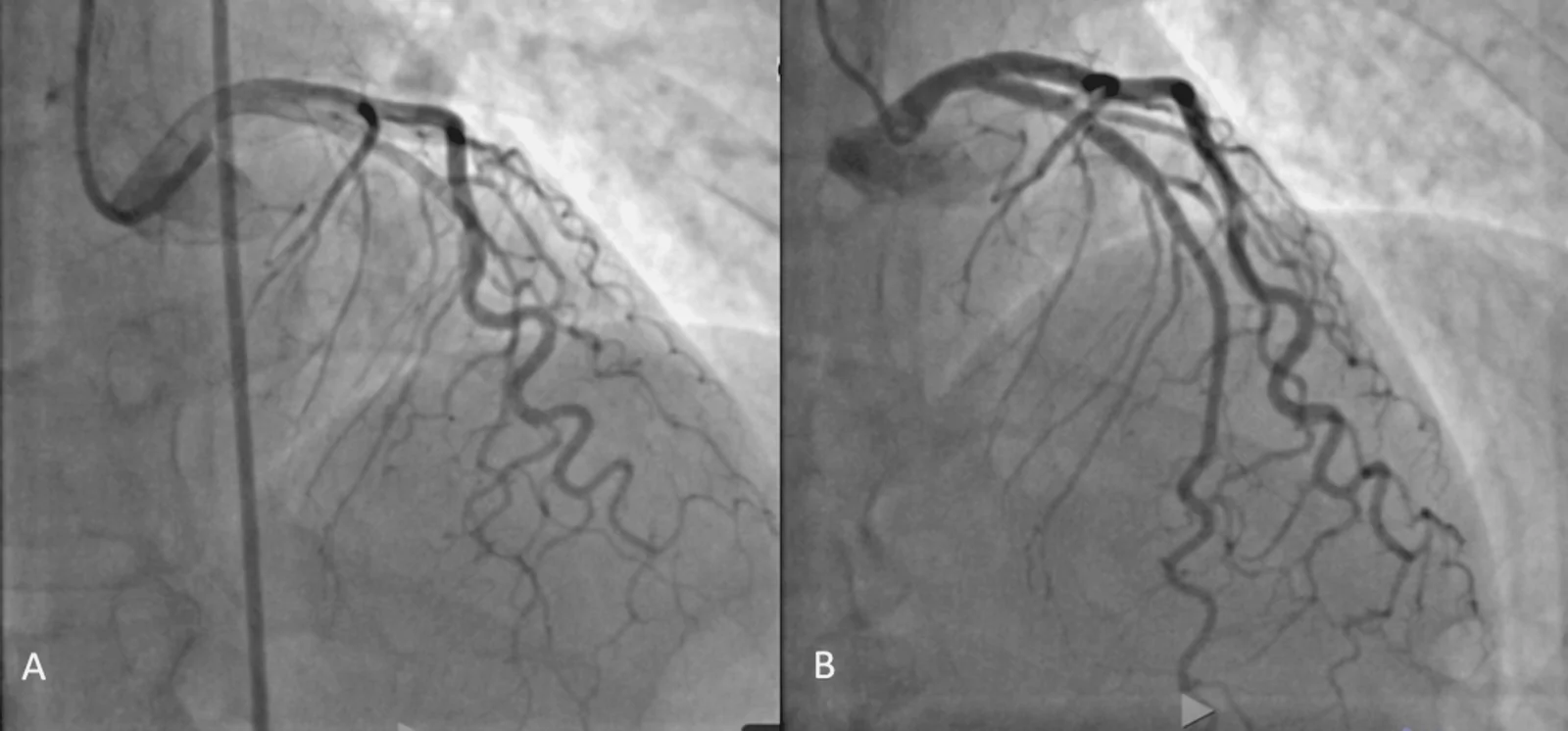
Left side (A): mid-to-distal left anterior descending LAD SCAD, Yip-Saw type 2; Right side (B): patent coronary arteries with resolution of spontaneous coronary artery dissection

A transthoracic echocardiogram showed a reduced left ventricular ejection fraction of 40% (Video 1), with apical ballooning in keeping with SICM. The diagnostic challenge in this case was distinguishing the underlying cause that represented the primary initiating event.

**Video 1:** Stress-induced cardiomyopathy Echo demonstrating reduced ejection fraction with apical ballooning.

The patient was subsequently managed conservatively. She was started on dual antiplatelet therapy, and a beta-blocker and angiotensin-converting enzyme inhibitor were added to her regimen. The patient remained hemodynamically stable and pain-free throughout her hospital course and was discharged in stable condition with close outpatient follow-up.

In conservatively managed SCAD, the optimal antiplatelet strategy remains uncertain given the infrequent presence of luminal thrombus and concern that prolonged DAPT may increase bleeding within the intramural hematoma. However, in the absence of an apparent high bleeding risk, our patient received a short four-week course of DAPT, consistent with expert recommendations supporting two to four weeks of DAPT during the acute healing phase, followed by aspirin monotherapy [5].

The patient remained adherent to prescribed medical therapy and tolerated treatment without medication-related adverse effects during follow-up. Repeat coronary angiography performed two months after the index presentation demonstrated complete restoration of the normal luminal caliber of the left anterior descending artery, with resolution of the previously observed diffuse narrowing (Figure 1, right panel). An interval echocardiogram also showed resolution of wall motion abnormalities and ventricular dysfunction (Video 2).

**Video 2:** Interval echocardiogram also showed resolution of wall motion abnormalities and ventricular dysfunction Echocardiogram demonstrating resolution of wall motion abnormalities and ventricular dysfunction

## Discussion

This case highlights the diagnostic and pathophysiologic intersection between SCAD and SICM, two conditions that disproportionately affect women and may present with overlapping clinical and imaging features. Emerging evidence suggests that these entities may be interrelated and have a bidirectional relationship in which either condition may precede or precipitate the other [3,4].

The concept of acute mechano-cardiac coronary artery disruption (AMCAD) provides a mechanistic framework linking these conditions. AMCAD proposes that abnormal myocardial contraction patterns, such as apical ballooning, can generate localized mechanical stress gradients that may transmit shear forces to the coronary vasculature, predisposing to dissection [6]. Within this model, SICM may act as the initiating event, with altered myocardial mechanics contributing to coronary wall disruption.

Conversely, SCAD may represent the primary event, with the acute ischemic insult and associated physiological stress triggering a secondary catecholamine surge capable of inducing Takotsubo-like ventricular dysfunction [3]. Importantly, the temporal relationship between these processes cannot be definitively established in this case, and both pathways remain biologically plausible.

The strong female predominance of both SCAD and SICM suggests a shared susceptibility rooted in sex-specific cardiovascular biology. Estrogen plays a central role in maintaining vascular integrity through the preservation of endothelial function, nitric oxide signaling, suppression of oxidative stress, and stabilization of the extracellular matrix, thereby exerting important cardioprotective and vascular protective effects [7].

Declining estrogen levels may therefore increase vulnerability to coronary artery wall disruption and abnormal vascular responses. This patient’s age of 48 years is consistent with the perimenopausal transition, a period characterized by fluctuating and declining estrogen levels, which may further contribute to increased susceptibility to both coronary artery dissection and stress-induced myocardial dysfunction.

In parallel, SICM is believed to involve excessive catecholamine stimulation leading to myocardial stunning, microvascular dysfunction, and transient ventricular systolic impairment. Experimental data further suggest that estrogen attenuates catecholamine-mediated myocardial injury through the modulation of β-adrenergic signaling, inflammatory pathways, and oxidative stress responses [8]. These overlapping hormonal and autonomic mechanisms may help explain the marked female predominance observed in both SCAD and SICM and support the concept of a shared stress-responsive cardiovascular phenotype.

The markedly elevated troponin level, peaking at 109,820 ng/L, is consistent with acute myocardial ischemia. BNP was elevated at 652 pg/mL; however, natriuretic peptide levels may be relatively lower than expected in individuals with elevated body mass index due to increased clearance and reduced secretion, which may underestimate the degree of myocardial wall stress. Thus, in a patient with BMI greater than 30, the measured BNP may not fully reflect the underlying hemodynamic burden [9].

Coronary angiography demonstrated a long, smooth, diffuse narrowing of the mid-to-distal LAD consistent with Yip-Saw Type 2 SCAD [10]. Although intracoronary imaging with OCT or IVUS was not performed, the characteristic angiographic appearance, persistence despite vasodilator administration, and complete spontaneous resolution on follow-up strongly support the diagnosis. Coronary vasospasm remains less likely given the lack of vasodilator response.

Taken together, the angiographic, biomarker, and clinical findings support SCAD-related ischemic myocardial injury as a major contributor, while the echocardiographic finding of apical ballooning remains in keeping with SICM. The relative contribution of each process cannot be definitively determined, reinforcing the bidirectional relationship between these entities.

However, we have integration of serial angiographic and echocardiographic follow-up demonstrating complete resolution of both the coronary abnormalities and ventricular dysfunction. Additionally, the patient was successfully managed conservatively without coronary stent implantation, resulting in full angiographic and functional recovery on follow-up evaluation. Limitations include the absence of intracoronary imaging with OCT or IVUS, which limited definitive confirmation of the dissection mechanism and prevented precise determination of the primary initiating event between SCAD and SICM.

Overall, this case underscores that SCAD and SICM may represent overlapping expressions of a shared stress-responsive cardiovascular phenotype in women, in which the initiating event is not always identifiable.

## Conclusions

SCAD and SICM may present with overlapping clinical features, making accurate diagnosis reliant on the careful integration of clinical findings, multimodality imaging, and biomarker data. This case highlights a potential bidirectional relationship between these entities, in which the inciting event may not always be readily identifiable.

Clinicians should maintain a high index of suspicion for concomitant SICM in patients presenting with SCAD, particularly when regional wall motion abnormalities extend beyond the perfusion territory of the affected coronary artery. Recognizing the possibility that mechanical stress associated with regional wall motion abnormalities may precipitate coronary artery dissection, or, conversely, that SCAD may trigger SICM, has important implications for diagnostic evaluation, clinical interpretation, and therapeutic decision-making in these complex presentations.
